# Development and Validation of a Depression Scale for Online Assessment: Cross-Sectional Observational Study

**DOI:** 10.2196/70689

**Published:** 2025-07-21

**Authors:** Minjeong Jeon, Hae-In Park, Yoorianna Son, Ji Won Hyun, Jin Young Park

**Affiliations:** 1 Department of Psychiatry, Yonsei University College of Medicine, Yongin Severance Hospital Yongin Republic of Korea; 2 Yonsei University College of Medicine Institute of Behavioral Science in Medicine Seoul Republic of Korea; 3 Yonsei University Health System Center for Digital Health, Yongin Severance Hospital Yongin Republic of Korea

**Keywords:** Depression Scale for Online Assessment, social media, scale development, depressive disorder, digital mental health, mobile phone

## Abstract

**Background:**

Despite increased awareness and improved access to care, depression remains underrecognized and undertreated, in part due to limitations in how current assessment tools capture emotional distress. Traditional depression scales often rely on fixed diagnostic language and may overlook the varied and evolving ways in which individuals express depressive symptoms—particularly in digital environments. Social media platforms have emerged as important spaces where people articulate psychological suffering through informal, emotionally charged language. These expressions, while nonclinical in appearance, may hold meaningful diagnostic value.

**Objective:**

This study aimed to develop and validate the Depression Scale for Online Assessment (DSO), a tool designed to capture ecologically valid expressions of depressive symptoms as articulated in digital contexts.

**Methods:**

A cross-sectional, observational study was conducted with a community sample of 1216 adults, from which 1151 valid responses were retained for analysis. The scale’s items were developed based on expert reviews and social media research. To identify the factor structure, exploratory factor analysis (EFA) was conducted on a randomly selected half of the sample (n=575), followed by confirmatory factor analysis on the remaining half (n=576) to validate the model. Internal consistency was assessed following the EFA, and convergent validity was examined by correlating each DSO factor score with established depression measures, including the Korean version of the Center for Epidemiologic Studies Depression Scale-Revised and the Patient Health Questionnaire-9.

**Results:**

EFA identified a 5-factor structure (ie, social disconnection, suicide risk, depressed mood, negative self-concept, and cognitive and somatic distress) that explained 66.53% of the total variance, indicating an acceptable level of explanatory power for a multidimensional psychological construct. confirmatory factor analysis indicated acceptable model fit (*χ*²_109_=403.5, *P*<.001; comparative fit index=0.96; Tucker-Lewis index=0.95; standardized root-mean-squared residual=0.03; root-mean-square error of approximation=0.07). The scale showed high internal consistency (total Cronbach α=0.95), and subscales were significantly correlated with the Center for Epidemiologic Studies Depression Scale-Revised (*r*=0.68-0.77) and the Patient Health Questionnaire-9 (*r*=0.64-0.74), supporting convergent validity.

**Conclusions:**

The DSO is a psychometrically sound and clinically relevant tool that captures both core and emerging expressions of depression. Its digital adaptability makes it especially useful for research and clinical practice in mobile and remote care settings.

## Introduction

### Background

Depression remains one of the most significant mental health challenges globally, affecting individuals in all aspects of life [1]. Beyond mood disturbances, depression impacts sleep, appetite, energy levels, cognitive functions, and interpersonal relationships. Despite increasing public awareness and improved access to care, depression continues to be underreported and undertreated across clinical and community settings. While widely used self-report tools have played an essential role in screening and research, questions remain about whether current assessment methods fully capture the evolving ways in which individuals experience and articulate emotional distress—particularly in light of changing communication habits and the growing influence of digital environments.

Scholars have long recognized that the effectiveness of traditional depression scales may be influenced not only by the content of their items but also by how symptoms are linguistically framed. For instance, Montgomery and Åsberg [2] noted that early depression rating scales were designed to reflect diagnostic criteria rather than to sensitively capture fluctuations in emotional states. In addition, standard symptom formulations—such as sadness or tearfulness—may not align with how some individuals, depending on cultural, generational, or gender norms, express psychological suffering. Hunt et al [3] found that conventional tools like the Beck Depression Inventory [4] missed a substantial number of depression cases in certain populations, prompting interest in how symptom expression shapes detection. These observations have led researchers to consider how more naturalistic or context-sensitive language might improve detection accuracy, particularly in light of recent shifts toward digital modes of emotional communication.

Social media, in particular, provides a venue where psychological distress is often expressed spontaneously and informally, using phrases and tones not typically found in legacy assessment tools. Platforms like Facebook (Meta), Twitter (subsequently rebranded as X), and Instagram (Meta) have become critical spaces where individuals express their emotions and connect with others. Studies have shown that depressive expressions and suicidal ideation are commonly observed on these platforms, making them valuable for the early detection of mental health risks [5-10]. While such expressions are not always clinically labeled, they may offer valuable insight into how emotional pain is experienced and described in everyday life.

Previous research has demonstrated that depressive and suicide-related language frequently appears in social networking system (SNS) content, offering real-time indicators of psychological distress. Numerous Korean studies, including those by Park et al [11], Song [12], Seo and Song [13], and Kim et al [14] have shown that SNS-based expressions—such as statements of hopelessness, isolation, or suicidal ideation—can be systematically analyzed to identify users at risk of depression. These findings suggest that digital expressions are not only reflections of individual distress but also potential signals for early detection. The digital footprint of SNS users, including language use and behavioral patterns, has been explored as a valuable predictor of depressive symptoms and subjective stress.

To address these challenges, this study focused on developing an assessment tool grounded in ecologically valid digital expressions of depression. Drawing on language frequently observed in social media platforms, the scale was designed to capture symptoms that may be underrepresented in conventional assessments. By incorporating brief, intuitive items that reflect how individuals express depressive feelings in everyday digital language use, the DSO seeks to enhance the ecological validity, emotional resonance, and contextual relevance of depression assessment in contemporary settings.

### Objectives

This study aims to develop and validate the Depression Scale for Online Assessment (DSO), a novel tool designed to capture the ways in which individuals experience and express depressive symptoms in digital environments. The proposed scale offers several key innovations. First, integration of social media–based language where items were generated based on expressions frequently used in social media posts by individuals conveying depressive emotions, enabling the scale to reflect forms of distress that may be overlooked by conventional tools. Second, ecological and practical design. With short, intuitive items optimized for digital delivery, the scale is suited for use in both clinical and research settings, particularly those involving mobile or web-based platforms.

By integrating clinically relevant content with ecologically grounded language, the DSO aims to provide a more nuanced and contextually appropriate approach to understanding and evaluating depression in the digital age.

## Methods

### Preliminary Item Development

In the development of a DSO, our research meticulously examined existing studies that investigated expressions commonly used by individuals displaying signs of depression on SNS. Our goal was to integrate the most current depressive trends into the scale, which led us to analyze studies conducted within the last 3 years that focused on depressive tendencies on SNS.

The foundational studies we considered include those by Zhu and colleagues [15], Seo and Song [13], Kim and team [14], Park and Yu [16], and Park et al [11]. These studies provided a wealth of keywords which we then used to create an initial pool of items for the scale. Delving into the specifics of each study, we found a range of keywords associated with depression. Zhu and colleagues [15] sourced keywords from Facebook groups centered around depression, highlighting the most frequently mentioned terms in posts, which spanned across personal identifiers, psychological or emotional states, temporal terms, and those related to pain or self-harm. Seo and Song [13] gathered Twitter data based on explicit phrases signifying a diagnosis or prescription of depression and conducted a co-occurrence network analysis based on the frequency of these terms appearing together. Kim and colleagues [14] created word clouds from tweets to distinguish between depressive and nondepressive sentiments, scaling words in size and frequency, with terms like “depression,” “people,” “lie,” and “suicide” being particularly prominent. Park and Yu [16] focused on Instagram posts related to “suicide” and “self-harm,” performing a frequency analysis to identify the most recurrent words. Finally, Park et al [11] used multiple social media sources to cluster expressions into categories aligned with the *DSM-5* (*Diagnostic and Statistical Manual of Mental Disorders* [Fifth Edition]) diagnostic criteria for depression.

The research team then selected the most relevant words pertaining to cognitive, emotional, and physical symptoms of depressive disorders, as well as those related to suicidality and emotionally distressing experiences frequently observed in previous SNS-based studies. These expressions were refined into brief, first-person statements beginning with “I feel...” or “I am...” to enhance emotional immediacy and consistency. Each item was deliberately designed to concise, generally limited to 3 word segments, in order to ensure high readability and rapid comprehension in mobile or web-based environments. This structure aligns with the practical demands of digital mental health screening, where user engagement and clarity are essential.

To verify the adequacy of our preliminary items and their distribution across various factors, we conducted a comparative analysis with established depression scales such as the Korean version of the Center for Epidemiologic Studies Depression Scale-Revised (K-CESD-R) [17] and the Patient Health Questionnaire-9 (PHQ-9) [18]. The final selection of items and their factor structure are detailed in Multimedia Appendix 1. The instructions for the scale prompt respondents to reflect on their past week and rate their experiences using a 5-point Likert scale, ranging from “0=Not at all” to “4=Very much so,” thereby facilitating a nuanced assessment of their depressive symptoms. The DSO was specifically crafted to offer convenience in a variety of settings, including mobile and web-based platforms, with an emphasis on ease of use and minimal participant burden. Consequently, we developed it as a self-report questionnaire with the aim of reducing the item number to approximately 20 items (less than 5 min completion time).

### Study Design

This study is an observational, cross-sectional study conducted through a web-based survey. The survey was administered to members of a web-based survey site, and data were collected between July and August 2023. The study adheres to the CHERRIES (Checklist for Reporting Results of Internet E-Surveys) guidelines [19] for reporting internet survey results.

### Sample Size Calculation

In factor analysis, absolute criteria for sample size are commonly defined as a sample size of 100 being considered poor, around 200 being fair, approximately 300 being good, around 500 being very good, and 1000 or more being regarded as excellent [20]. Regarding the ratio of sample size to the number of measured variables, different scholars propose varying recommendations. However, it is generally advised to have a sample size at least 20 times the number of factors to be extracted to ensure stable results [21]. Based on these criteria, the target number of participants for this study was set to exceed 1000.

### Participants

Participants eligible for inclusion in the study were adults aged 19 years and older, with quotas set to ensure equal distribution by age, region, and gender. Exclusion criteria included individuals who did not complete the survey or provided responses that appeared insincere based on response patterns. A total of 1216 individuals completed the survey. After excluding 65 cases due to incomplete or insincere responses, data from 1151 participants were retained for final analysis.

### Setting

This study recruited participants from a web-based panel managed by dataSpring Inc, a professional survey company specializing in surveys and research services. The company maintains a diverse participant pool through ongoing recruitment across various demographics, including age, gender, and region, and uses rigorous identity verification processes.

For this study, participants aged 19 years and older residing in different provinces of South Korea were selected using quota sampling based on age group, region, and gender. Eligible participants within these quota groups were randomly invited through an invitation link containing a brief study description and eligibility criteria. To encourage participation, monetary incentives were provided upon survey completion.

Participation was entirely voluntary, and participants had the option to withdraw at any point before submitting their responses without any penalty. They could also review and modify their answers before final submission, ensuring flexibility. Survey responses were automatically recorded and securely stored in a database, and only fully completed questionnaires were included in the analysis.

### Content Validity

Through an extensive literature review, a comprehensive DSO was developed, encompassing 44 items across 5 key factors (Multimedia Appendix 1). To ensure clinical relevance and content validity, the preliminary item pool was reviewed by a panel of 17 experts, including psychiatrists and clinical psychologists. These experts evaluated each item’s semantic clarity, and appropriateness for assessing depressive symptoms. Based on their feedback, items with low relevance, redundancy, or ambiguity were eliminated or revised, resulting in a final set of 20 items.

### Statistical Analysis

First, exploratory factor analysis (EFA) was conducted to identify the factors of the DSO. To ensure methodological rigor and reduce overfitting risk, this dataset was randomly divided into 2 independent subsamples. EFA was conducted on one half (n=575), and confirmatory factor analysis (CFA) was conducted on the other half (n=576). EFA was used to identify the underlying factor structure of the DSO. The suitability of the data was confirmed using the Kaiser–Meyer–Olkin (KMO) test and Bartlett test of sphericity. The KMO measure assesses sampling adequacy for factor analysis, with values closer to 1 indicating more reliable results. Communality refers to the proportion of variance in each item that is explained by the extracted factors, with low values (<0.4) suggesting limited relevance to the overall structure. Factor extraction was performed using principal axis factoring with promax (oblique) rotation, which allows for correlations among factors and is appropriate for the theoretically related dimensions of depression. The number of factors to retain was determined using multiple criteria, including eigenvalues (>1.0), scree plot inspection, theoretical interpretability, and parallel analysis. Parallel analysis (principal axis factoring, 100 iterations), conducted using the fa.parallel function in the psych package in R (R Core Team). Parallel analysis has been recommended in the literature as a more accurate and robust method for factor retention than the traditional Kaiser criterion [22,23]. After factor extraction, internal consistency for each factor was assessed using Cronbach α to evaluate the reliability of the subscales. CFA was subsequently conducted to verify the fit of the factor structure derived from the EFA. The analysis was performed using the “lavaan” package in R. Model fit was assessed using several commonly used indices, including the chi-square test, comparative fit index (CFI), Tucker-Lewis index (TLI), root-mean-square error of approximation (RMSEA), and standardized root-mean-square residual (SRMR). According to established guidelines, CFI and TLI values above 0.90 indicate acceptable fit (with >0.95 considered excellent), RMSEA values below 0.08 reflect reasonable fit (with <0.05 excellent), and SRMR values below 0.08 are considered desirable. Pearson correlation coefficients were calculated to examine concurrent validity against the Center for Epidemiologic Studies Depression Scale-Revised (CESD-R) and PHQ-9. Cronbach α was used to evaluate internal consistency reliability for each subscale and the overall instrument. All statistical analyses, including EFA, CFA, and reliability testing, were performed using R (version 4.5.0).

**Figure 1:**
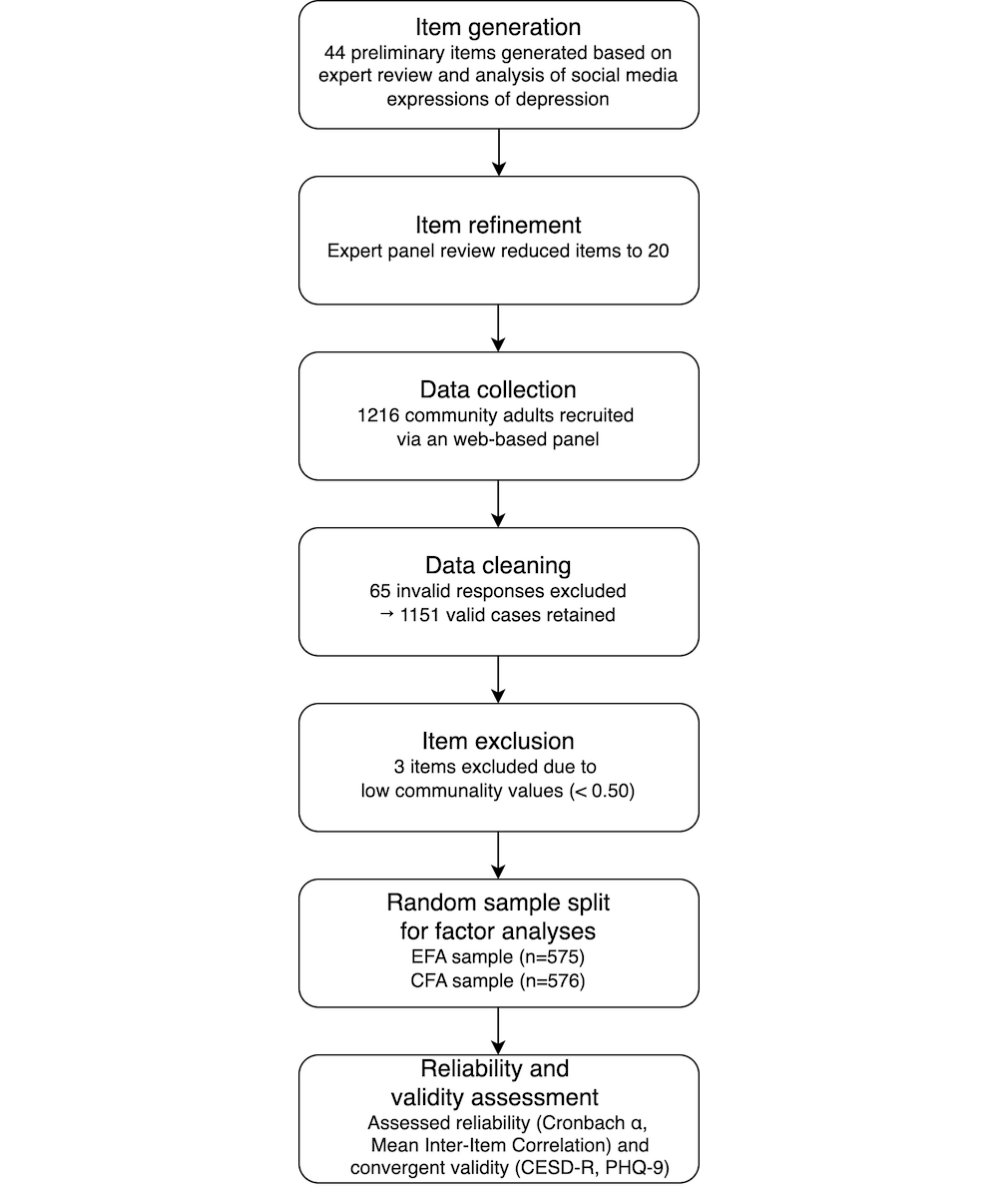
Study procedure and validation workflow, including item generation, data collection, factor analyses, reliability testing, and validity assessment. CESD-R: the Center for Epidemiologic Studies Depression Scale-Revised; CFA: confirmatory factor analysis; EFA: exploratory factor analysis; PHQ-9: the Patient Health Questionnaire-9.

A visual summary of the scale development and validation process is provided in Figure 1, outlining the key stages from item generation to final psychometric evaluation.

### Ethical Considerations

This study was approved by the institutional review board (IRB) of Yongin Severance Hospital (IRB 2023-0153-005). Before obtaining consent, participants were informed of the study’s purpose, procedures, the voluntary nature of participation, and their right to withdraw at any time. All personal information collected was anonymized, encrypted, and securely managed. In compliance with the Bioethics and Safety Act, the data will be destroyed 3 years after the study’s completion. In accordance with the approved IRB protocol, participants who completed the survey received 1500 points through a contracted web-based survey agency (DataSpring), which could be redeemed as a cash transfer, gift card, or donation. Participants were also informed that they could withdraw from the study at any time without penalty. To address potential psychological distress caused by responding to sensitive items, emergency contact information was provided, and participants were guided to a nearby emergency room if necessary. Any adverse events were to be promptly reported and managed according to the IRB-approved protocol.

### Measure and Scale

The following scale was used to verify the convergent validity with the DSO.

#### PHQ-9

PHQ-9 [18] is a depressive disorder module of the Primary Care Evaluation of Mental Disorder developed for the diagnosis of common mental disorders in primary medical institutions, consisting of 9 items (depressed mood, loss of interest, sleep disturbance, appetite changes, psychomotor changes, fatigue, low self-esteem, concentration problems, and suicidal thoughts) that correspond to the *DSM-IV* (*Diagnostic and Statistical Manual of Mental Disorders* [Fourth Edition]) diagnostic criteria for major depressive disorders, and to find out how often these problems have been experienced over the past 2 weeks using the Korean version of PHQ-9, which was translated and studied in Korea in 2007 and confirmed its validity and reliability [24]. The response is evaluated on a 4-point scale, and the score range consists of 0-27. A sum of scores of 10 or more is evaluated as having major depressive disorders. This study was conducted to confirm that the depression scale developed in this study and PHQ-9, which is a depression screening tool with high reliability and validity, showed high coexistence validity.

#### K-CESD-R

K-CESD-R [17] is a depression scale developed by Eaton and Smith [17] and validated by Lee et al [25] with the K-CESD-R, reflecting the duration and symptoms of major depressive abstraction in accordance with the revision of the diagnostic criteria to *DSM-IV*. It consists of a total of 20 questions, and is instructed to respond to how often you have felt this feeling over the past 2 weeks, and is evaluated on a 5-point scale. The higher the score, the higher the degree of depression, and if the score is 13 or higher, it is evaluated as having a depressive disorder. This study was conducted to confirm that the depression scale developed in this study and the K-CESD-R, a depression screening tool with high reliability and validity, show high coexistence validity.

## Results

### Demographics

Table 1 presents data on the sex, age, and educational background of the respondents in the questionnaire used to analyze the study results.

**Table 1:** Sociodemographic characteristics of the study participants (N=1151).

Characteristic		Value, n (%)
**Sex**		
	Male	579 (50.3)
	Female	572 (49.7)
**Age (y)**		
	20-29	239 (20.8)
	30-39	199 (17.3)
	40-49	237 (20.6)
	50-59	275 (23.9)
	>60	201 (17.5)
**Education**		
	High school graduate	180 (15.64)
	Graduate	769 (66.81)
	Master’s degree	71 (6.17)
	Doctor’s degree	12 (1.04)
	Unidentified	119 (10.34)

### Factor Analysis

Out of the initial 20 items, 3 were excluded before EFA because they did not meet the communality threshold of 0.50. Communality values below this threshold indicate that an item does not share sufficient variance with the extracted factors, making it unsuitable for factor analysis. Table 2 presents an overview of the item reduction process and the rationale for item retention.

**Table 2:** Depression Scale for Online Assessment item development summary.

Stage	Number of items	Exclusion criteria	Notes
Initial item pool	44	Redundancy, unclear wording, lack of relevance to depression	Items were generated based on *DSM-5*^a^, clinical expertise, and social media expressions
After expert review	20	Low content validity, overlap with other items	Expert panel reviewed items for clarity, coverage, and cultural appropriateness
After EFA^b^ preparation (final items retained)	17	Communality <0.5	Items with weak statistical performance were excluded

^a^DSM-5: Diagnostic and Statistical Manual of Mental Disorders (Fifth Edition).

^b^EFA: exploratory factor analysis.

The results of the KMO test and Bartlett test of sphericity were confirmed among the 17 items. In this analysis, the KMO value of the scale was found to be good at 0.95, and Bartlett sphericity was also statistically significant (*χ*^2^_136_=6961.9, *P*<.001), indicating the adequacy of the data for factor analysis.

Parallel analysis supported the retention of 5 factors, as the eigenvalues from the actual data exceeded those derived from randomly generated datasets across 100 iterations. Accordingly, EFA extracted five distinct factors:

1. Social disconnection, consisting of 4 items (eigenvalue=3.27, variance explained=19.24%);

2. Depressed mood, consisting of 4 items (eigenvalue=2.69, variance explained=15.64%);

3. Suicide risk, consisting of 2 items (eigenvalue=2.06, variance explained=12.11%);

4. Negative self-concept, consisting of 3 items (eigenvalue=1.69, variance explained=9.91%);

5. Cognitive and somatic distress, consisting of 4 items (eigenvalue=1.64, variance explained=9.64%).

Together, these 5 factors explained 66.53% of the total variance, indicating a robust level of explanatory power for the underlying construct of depression symptoms measured by the DSO (Table 3). Most items loaded clearly onto single factors. However, 2 items—“I hate myself” (C31) and “My memory has worsened” (C36)—showed meaningful cross-loadings. C35 loaded strongly on the negative self-concept factor but also exhibited secondary loading on the social disconnection factor. C36 showed nearly equal loadings on both the negative self-concept and cognitive and somatic distress factors (0.40), suggesting a conceptual overlap between memory difficulties and core depressive self-evaluation. In addition, P29 (“I feel slowed down”) demonstrated a modest secondary loading (0.28) on the negative self-concept factor but was primarily associated with the cognitive and somatic distress factor (0.42). These items were retained based on theoretical rationale and their relevance to the clinical construct of depression. This decision aligns with previous psychometric guidelines that support retaining cross-loading items when they contribute meaningfully to construct representation and content validity [26].

**Table 3:** Exploratory factor analysis on Depression Scale for Online Assessment (KMO=0.947, Bartlett χ^2^_136_=6961.9, *P*<.001).

Item number	Communality	Social disconnection	Depressed mood	Suicide risk	Negative self-concept	Cognitive and somatic distress
E14	0.94	0.94	–0.02	–0.02	–0.07	0.04
E10	0.94	0.76	0.16	0.01	0.01	–0.04
E16	0.96	0.68	–0.02	0.10	0.13	0.08
E41	0.96	0.54	0.06	0.01	0.19	0.05
S26	0.91	–0.01	0.01	0.88	0.08	–0.03
S8	0.90	–0.003	0.01	0.80	–0.10	0.09
C25	0.96	0.20	0.16	0.25	0.42	–0.03
C35	0.95	0.17	0.15	0.21	0.53	–0.01
C31	0.96	0.31	0.08	0.22	0.40	0.03
C6	0.95	0.02	0.60	–0.07	0.21	0.22
C23	0.96	0.06	0.48	0.02	0.23	0.24
M1	0.95	–0.01	0.84	0.04	–0.06	0.001
M5	0.96	0.17	0.70	0.09	–0.04	–0.06
C36	0.95	0.001	0.06	0.02	0.40	0.40
P29	0.97	0.12	0.13	0.03	0.28	0.42
P17	0.92	0.17	–0.01	0.11	–0.03	0.54
P7	0.95	0.08	0.12	0.09	–0.14	0.62
Eigenvalue	—^a^	3.27	2.66	2.06	1.69	1.64
Variance explanation power, %	—	19.24	15.64	12.11	9.91	9.64
Cumulative variance explanatory power, %	—	19.24	34.87	46.98	56.89	66.53

^a^Not applicable.

The internal consistency reliability of each factor in the DSO was evaluated using Cronbach α and the mean interitem correlation (Table 4). The social disconnection factor showed excellent internal consistency (Cronbach α=0.90, *r̄*=0.69). The depressed mood factor also demonstrated strong reliability (Cronbach α=0.89, *r̄*=0.66). For the suicide risk factor, which consisted of only 2 items, we relied on the mean interitem correlation (*r̄*=0.72), which fell within the optimal range (0.70-0.90), suggesting a high degree of internal consistency despite the limited item count. Cronbach α for this factor was 0.83. The negative self-concept factor yielded Cronbach α=0.89 and *r̄*=0.73. The cognitive and somatic distress factor demonstrated acceptable internal consistency (Cronbach α=0.78, *r̄*=0.47). The overall internal consistency of the 17-item DSO was excellent (Cronbach α=0.95, *r̄*=0.52), indicating strong reliability across both subscales and the total scale.

**Table 4:** Subsets of Depression Scale for Online Assessment and their reliability.

Factor	Item numbers	Number of items	Cronbach α	Mean interitem correlation
Social disconnection	14, 10, 16, 41	4	0.90	0.69
Depressed mood	6, 23, 1, 5	4	0.89	0.66
Suicide risk	26, 8	2	0.83	0.72
Negative self-concept	25, 35, 31	3	0.89	0.73
Cognitive and somatic symptom	36, 29, 17, 7	4	0.78	0.47
Total		17	0.95	0.52

**Figure 2:**
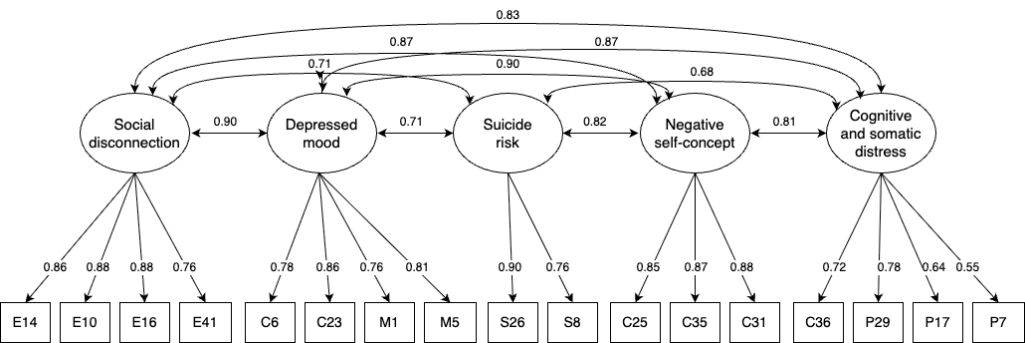
Confirmatory factor analysis model of the Depression Scale for Online Assessment, illustrating the final 5-factor structure.

CFA was conducted on an independent subsample (n=576) to validate the 5-factor structure derived from the EFA (Figure 2). The analysis was performed using the lavaan package in R. Model fit was evaluated using multiple indices: the chi-square test, CFI, TLI, RMSEA, and SRMR. According to the established benchmarks, CFI and TLI values above 0.90 are considered acceptable (with >0.95 deemed excellent), RMSEA values below 0.08 indicate reasonable fit (with <0.05 excellent), and SRMR values below 0.08 are desirable.

The model demonstrated good overall fit: CFI=0.96, TLI=0.95, and SRMR=0.03, all within the excellent range. The RMSEA was 0.07 (90% CI 0.06-0.08), which falls within the acceptable range, although not excellent. While the chi-square test was significant (*χ*²_109_=403.5, *P*<.001), this result is expected given the large sample size, as this statistic is known to be highly sensitive to sample size. Taken together, the findings support the adequacy of the 5-factor model and confirm the factorial validity of the DSO.

### Convergent Validity Analysis

Pearson correlation coefficient analyses were conducted to evaluate the convergent validity of the DSO subscales by examining their relationships with established depression scales (CESD-R and PHQ-9). All 5 subscales of the DSO showed strong and statistically significant positive correlations with both the CESD-R (*r*=0.68-0.77, *P*<.001) and the PHQ-9 (*r*=0.64-0.74, *P*<.001), indicating good convergent validity (Table 5). These findings suggest that the DSO subdimensions align well with established measures of depressive symptomatology.

**Table 5:** Convergent validity of Depression Scale for Online Assessment subscales with the Center for Epidemiologic Studies Depression Scale-Revised and the Patient Health Questionnaire-9.

DSO^a^ factor	CESD-R^b^			PHQ-9^c^	
	Pearson correlation coefficient (*r*)	*P* value	Pearson correlation coefficient (*r*)		*P* value
Social disconnection	0.74	<.001	0.71		<.001
Depressed mood	0.76	<.001	0.74		<.001
Suicide risk	0.68	<.001	0.64		<.001
Negative self-concept	0.77	<.001	0.73		<.001
Cognitive and somatic symptom	0.72	<.001	0.73		<.001

^a^DSO: Depression Scale for Online Assessment.

^b^CESD-R: Centre for Epidemiological Studies in Depression Revised.

^c^PHQ-9: the Patient Health Questionnaire-9.

## Discussion

### Principal Findings

This study presents the development and validation of the DSO, a novel self-report measure of depressive symptoms that is optimized for digital environments by incorporating linguistic features frequently found in social media expressions, as well as traditional clinical symptomatology. Based on a large community sample (N=1151), exploratory and confirmatory factor analyses supported a 5-factor structure encompassing social disconnection, suicide risk, depressed mood, negative self-concept, and cognitive and somatic distress. The model explained 66.53% of the total variance, indicating robust structural validity. CFA demonstrated good fit indices (CFI=0.96, TLI=0.95, and SRMR=0.03), with RMSEA=0.07 falling within the acceptable range. Although the chi-square statistic was significant, this is consistent with expectations for large samples. Reliability was excellent for the overall scale (Cronbach α=0.91), and acceptable to strong for all subscales. Convergent validity was supported by high correlations with the CESD-R and PHQ-9.

We acknowledge that some items in the final DSO scale exhibited cross-loadings. For example, the items “I hate myself” and “My memory has been bad” exhibited cross-loadings between factors—specifically, “negative self-concept” and both “social disconnection” and “cognitive and somatic distress,” respectively. These overlaps were not treated as statistical artifacts but interpreted as theoretically coherent intersections that reflect the multifaceted nature of depression. Such cross-loadings are common in depression research. Given the inherently overlapping nature of depressive symptoms, they may even be expected rather than considered anomalies. This is because depressive symptomatology is increasingly understood not as isolated constructs, but as clusters of co-occurring symptoms reflecting affective, cognitive, somatic, and interpersonal dimensions. This complexity is reflected in numerous diagnostic and epidemiological studies that show substantial comorbidity and symptom clustering within depressive presentations. For example, Romera et al [27] reported overlapping factor loadings in primary care samples of patients with depression. Recent work by Fried and Nesse [28] and Borsboom [29] also suggest that depressive symptoms form interconnected networks rather than discrete units, which supports the notion that cross-loadings reflect meaningful overlap rather than error.

Rather than treating these overlaps as statistical anomalies, we considered them theoretically and clinically meaningful. For instance, memory difficulties are often correlated with self-perceived cognitive impairment and self-worth. Lower self-esteem has been linked to poorer episodic memory, especially when individuals view themselves as cognitively limited [30]. Self-dislike, meanwhile, may reflect internalized stigma and feelings of social disconnection. Research suggests that self-concept confusion and self-critical thinking are associated with internalized stigma and internalizing symptoms such as depression [31]. In addition, loneliness and social disconnection have been found to influence self-evaluative brain processes, contributing to more negative self-perceptions [32]. Methodologists have cautioned against the automatic deletion of cross-loading items when they carry clear conceptual weight, and their presence can enhance the content validity and clinical interpretability of multidimensional psychological measures. This perspective aligns with our approach to scale refinement. We therefore interpreted such patterns as a reflection of the syndrome’s clinical complexity rather than a methodological flaw. Previous psychometric literature also supports the retention of such items when they reflect theoretically grounded overlaps between constructs [26,33].

While the suicide risk factor in the DSO comprises only 2 items, we retained it based on both empirical justification and clinical urgency. Although 2-item factors are often critiqued for limited reliability, the suicide risk subscale demonstrated a strong mean interitem correlation (*r*=0.72) and an acceptable Cronbach α=0.83, both exceeding commonly accepted psychometric thresholds. Furthermore, suicidal ideation and self-harming tendencies are critical indicators of depression severity and treatment urgency. Removing such a factor could diminish the clinical completeness of the scale.

### Digital Relevance and Clinical Implications

The DSO was designed to reflect how depression is experienced and expressed in contemporary digital environments. Its item pool was informed by both traditional diagnostic frameworks and linguistic patterns observed in social media expressions. This digital adaptability makes the DSO particularly suited for use in mobile health applications, digital triage, and remote clinical monitoring. In clinical settings, it may serve as a rapid preliminary screener or provide contextual depth when used alongside conventional tools, especially where digital self-reporting is prioritized. The brevity and mobile compatibility of the DSO allow for efficient integration into telehealth platforms or chatbot-based interventions. Furthermore, the DSO’s 5-factor model captures all core domains of major depressive disorder as defined by the *DSM-5-TR* (*Diagnostic and Statistical Manual of Mental Disorders, Fifth Edition, Text Revision*), including affective, cognitive, and somatic symptoms, as well as critical risk indicators such as suicidality [34]. This ensures that the tool remains clinically grounded while being digitally adaptable.

In addition to its stand-alone utility, the DSO’s strong convergent validity and digital adaptability suggest that it can complement conventional tools in clinical settings. It may enrich diagnostic clarity by offering nuanced digital symptom profiles when used alongside established depression scales.

### Digital Public Health Implementation and Integration

Given the DSO’s strong validity and digital readiness, it holds promise for integration into public mental health strategies. Its compatibility with web-based platforms enables scalable screening in large populations, which aligns with current recommendations for using mobile technologies to improve access and quality in digital mental health services [35]. The DSO could also be embedded into digital health systems for continuous monitoring or real-time risk detection, particularly among individuals expressing depressive ideation on social media or other digital platforms. Recent studies suggest that predictive algorithms and self-report screening tools can be effectively integrated for early identification of mental health concerns in digital spaces [36,37]. In addition, the DSO’s concise structure and evidence-based item content make it suitable for use in mental health apps, similar to other tools evaluated for their clinical utility in self-monitoring and symptom feedback [38]. These capabilities position the DSO as a practical and scalable tool for early detection, triage, and intervention within digital public health frameworks.

### Limitations and Future Directions

While this study provides robust initial evidence for the validity and reliability of the DSO, several important directions remain for future research. First, as the current validation was conducted with a nonclinical community sample, it is necessary to replicate the findings in clinically diagnosed populations. Verifying the factor structure, sensitivity, and specificity of the DSO among individuals with major depressive disorder will be critical to determine its diagnostic and therapeutic utility. Second, for the scale to be used in clinical decision-making, interpretive benchmarks must be established. Future studies should explore optimal cutoff scores using receiver operating characteristic analysis to distinguish between individuals with and those without depression with high accuracy. Third, both the item development and validation procedures were conducted in Korean using Korean-language instruments, limiting cross-cultural generalizability. Expanding the DSO into multilingual versions and conducting cross-cultural validation studies would be essential to ensure its applicability in diverse linguistic and cultural contexts. Finally, as this was a cross-sectional study, longitudinal research is needed to assess the scale’s temporal stability and predictive validity. Future investigations should examine whether DSO scores can predict symptom trajectories, treatment response, or relapse in depressive disorders.

### Conclusion

The DSO is a psychometrically sound, clinically relevant, and digitally responsive measure of depressive symptoms. Its validated 5-factor structure reflects core and emerging expressions of depression in contemporary contexts. The DSO offers unique utility for both research and practice, particularly in digital health and remote care environments, and complements existing tools by addressing a critical gap in modern depression assessment.

## Appendix

Multimedia Appendix 1 Items for the development of the scale.

## Abbreviations

CHERRIES: Checklist for Reporting Results of Internet E-Surveys
CESD-R: the Center for Epidemiologic Studies Depression Scale-Revised
CFA: confirmatory factor analysis
CFI: comparative fit index
DSM-5: Diagnostic and Statistical Manual of Mental Disorders, Fifth Edition
DSM-5-TR: Diagnostic and Statistical Manual of Mental Disorders, Fifth Edition, Text Revision
DSM-IV: Diagnostic and Statistical Manual of Mental Disorders, Fourth Edition
DSO: Depression Scale for Online Assessment
EFA: exploratory factor analysis
IRB: Institutional Review Board
K-CESD-R: Korean version of the Centre for Epidemiological Studies in Depression Revised
KMO: Kaiser–Meyer–Olkin
PHQ-9: the Patient Health Questionnaire-9
RMSEA: root mean-square-error of approximation
SNS: social networking system
SRMR: standardized root-mean-squared residual
TLI: Tucker-Lewis index

## Data Availability

The datasets generated and analyzed during the current study are not publicly available due to institutional review board restrictions but are available from the corresponding author on reasonable request.

## References

[1] (2021). Global burden of disease study 2019. Global Burden of Disease Collaborative Network.

[2] Montgomery SA, Asberg M (1979). A new depression scale designed to be sensitive to change. Br J Psychiatry.

[3] Hunt M, Auriemma J, Cashaw ACA (2003). Self-report bias and underreporting of depression on the BDI-II. J Pers Assess.

[4] Beck AT, Steer RA, Brown G (1996). Beck Depression Inventory (2nd ed.) Manual.

[5] Keles B, McCrae N, Grealish A (2019). A systematic review: the influence of social media on depression, anxiety and psychological distress in adolescents. International Journal of Adolescence and Youth.

[6] Eichstaedt JC, Smith RJ, Merchant RM, Ungar LH, Crutchley P, Preoţiuc-Pietro D, Asch DA, Schwartz HA (2018). Facebook language predicts depression in medical records. Proc Natl Acad Sci U S A.

[7] De Choudhury M, Gamon M, Counts S, Horvitz E (2021). Predicting depression via social media. ICWSM.

[8] Figuerêdo JSL, Maia ALL, Calumby RT (2022). Early depression detection in social media based on deep learning and underlying emotions. Online Social Networks and Media.

[9] Liu D, Feng XL, Ahmed F, Shahid M, Guo J (2022). Detecting and measuring depression on social media using a machine learning approach: systematic review. JMIR Ment Health.

[10] Seabrook EM, Kern ML, Fulcher BD, Rickard NS (2018). Predicting depression from language-based emotion dynamics: longitudinal analysis of facebook and twitter status updates. J Med Internet Res.

[11] Park SJ, Lee S, Kim WJ (2022). A deep learning-based depression trend analysis of Koreans on social media. J Korean Soc Inf Manag.

[12] Song T (2015). Analysis of depression status and risk factors among Korean adolescents using social big data. Ministry of Health and Welfare Issue & Focus.

[13] Seo HR, Song M (2019). An analysis of the discourse topics of users who exhibit symptoms of depression on social media. Korean Soc Inf Manag.

[14] Kim Y, Moon J, Oh U (2020). Analysis and recognition of depressive emotion through NLP and machine learning. J Converg Cult Technol.

[15] Zhu YJ, Kim DH, Lee C (2019). Investigating major topics through the analysis of depression-related facebook group posts. Korean Soc Libr Inf Sci.

[16] Park S, Yu K (2021). Analysis of instagram posts related to self-injury and suicide using text mining. Korean J Couns Psychother.

[17] Eaton WW, Smith C, Maruish ME (2004). Center for epidemiologic studies depression scale: review and revision (CESD and CESD-R). The Use of Psychological Testing for Treatment Planning and Outcomes Assessment: Instruments for Adults.

[18] Kroenke K, Spitzer RL, Williams JBW (2002). The PHQ-15: validity of a new measure for evaluating the severity of somatic symptoms. Psychosom Med.

[19] Eysenbach G (2004). Improving the quality of web surveys: the checklist for reporting results of internet E-surveys (CHERRIES). J Med Internet Res.

[20] Comrey AL, Lee HB (1992). A First Course in Factor Analysis, 2nd edition.

[21] Arrindell WA, van der Ende J (1985). An empirical test of the utility of the observations-to-variables ratio in factor and components analysis. Applied Psychological Measurement.

[22] Horn JL (1965). Arationale and test for the number of factors in factor analysis. Psychometrika.

[23] Hayton JC, Allen DG, Scarpello V (2004). Factor retention decisions in exploratory factor analysis: a tutorial on parallel analysis. Organizational Research Methods.

[24] Choi H, Choi J, Park K, Joo K, Ga H, Ko H, Kim SR (2007). Standardization of the Korean version of patient health questionnaire-9 as a screening instrument for major depressive disorder. J Korean Acad Fam Med.

[25] Lee S, Oh ST, Ryu SY, Jun JY, Lee K, Lee E, Park JY, Yi SW, Choi W-J (2016). Validation of the Korean version of Center for Epidemiologic Studies Depression Scale-Revised(K-CESD-R). Korean J Psychosom Med.

[26] Worthington RL, Whittaker TA (2006). Scale development research: a content analysis and recommendations for best practices. The Counseling Psychologist.

[27] Romera I, Delgado-Cohen H, Perez T, Caballero L, Gilaberte I (2008). Factor analysis of the zung self-rating depression scale in a large sample of patients with major depressive disorder in primary care. BMC Psychiatry.

[28] Fried EI, Nesse RM (2015). Depression sum-scores don't add up: why analyzing specific depression symptoms is essential. BMC Med.

[29] Borsboom D (2017). A network theory of mental disorders. World Psychiatry.

[30] Pinard F, Vanneste S, Taconnat L (2021). Self-esteem effect on recall and recognition in episodic memory, in young and older adults. Exp Aging Res.

[31] Spoth R, Trudeau L, Shin C, Randall GK, Mason WA (2019). Testing a model of universal prevention effects on adolescent relationships and marijuana use as pathways to young adult outcomes. J Youth Adolesc.

[32] Staats S, van der Valk IE, Meeus WHJ, Branje SJT (2018). Longitudinal transmission of conflict management styles across inter-parental and adolescent relationships. J Res Adolesc.

[33] DeVellis RF (2016). Scale Development: Theory and Applications.

[34] American Psychiatric Association (2022). Diagnostic and Statistical Manual of Mental Disorders.

[35] Torous J, Jän Myrick K, Rauseo-Ricupero N, Firth J (2020). Digital mental health and COVID-19: using technology today to accelerate the curve on access and quality tomorrow. JMIR Ment Health.

[36] Chancellor S, De Choudhury M (2020). Methods in predictive techniques for mental health status on social media: a critical review. NPJ Digit Med.

[37] Birnbaum ML, Ernala SK, Rizvi AF, Arenare E, R Van Meter A, De Choudhury M, Kane JM (2019). Detecting relapse in youth with psychotic disorders utilizing patient-generated and patient-contributed digital data from Facebook. NPJ Schizophr.

[38] Wasil AR, Venturo-Conerly KE, Shingleton RM, Weisz JR (2019). A review of popular smartphone apps for depression and anxiety: assessing the inclusion of evidence-based content. Behav Res Ther.

