# Supplementary material for: Development and Validation of a Depression Scale for Online Assessment: Cross-Sectional Observational Study
Source: J Med Internet Res. 2025 Jul 21;27:e70689. doi: 10.2196/70689 (PMC12337799; doi:10.2196/70689)
Supplement: Multimedia Appendix 1 [file jmir_v27i1e70689_app1.docx]

| Construct and variable | | Item content | Number of items |
| --- | --- | --- | --- |
| **Mood** | |  | 12 |
|  | M1 | I feel depressed. |  |
|  | M5 | I feel sad. |  |
|  | M9 | I feel anxious. |  |
|  | M11 | I am restless. |  |
|  | M19 | I feel okay. |  |
|  | M22 | I get irritated easily. |  |
|  | M24 | I feel happy. |  |
|  | M27 | I am struggling. |  |
|  | M30 | I am in pain. |  |
|  | M32 | I cry more often. |  |
|  | M37 | I feel confused. |  |
|  | M38 | I am upset. |  |
| **Cognitive** | |  | 14 |
|  | C2 | I feel guilty. |  |
|  | C6 | I have no motivation. |  |
|  | C12 | My self-esteem has dropped. |  |
|  | C15 | I feel worthless. |  |
|  | C20 | I can not concentrate. |  |
|  | C23 | I have no energy. |  |
|  | C25 | I am a failure. |  |
|  | C28 | I am being punished. |  |
|  | C31 | I hate myself. |  |
|  | C33 | I do not enjoy anything. |  |
|  | C34 | I am a bad person. |  |
|  | C35 | I feel hopeless. |  |
|  | C36 | My memory has been bad. |  |
|  | C39 | I am stressed out. |  |
| **Physical** | |  | 7 |
|  | P3 | I am burned out. |  |
|  | P7 | I am eating more or less. |  |
|  | P13 | I have gained or lost weight. |  |
|  | P17 | I have been sleeping more or less. |  |
|  | P21 | I feel tired than usual. |  |
|  | P29 | I feel slowed down. |  |
|  | P42 | I feel sick |  |
| **Suicide or self-harm** | |  | 3 |
|  | S8 | I want to hurt myself. |  |
|  | S18 | I want to commit suicide. |  |
|  | S26 | I want to die. |  |
| **Others** | |  | 8 |
|  | E4 | I need comfort. |  |
|  | E10 | I feel lonely. |  |
|  | E14 | I feel alone. |  |
|  | E16 | I feel left out. |  |
|  | E40 | I am all alone. |  |
|  | E41 | People wear me out. |  |
|  | E43 | I worry about my health. |  |
|  | E44 | I am sensitive. |  |
